# Effect of Two Different Doses of Vitamin D Supplementation on Metabolic Profiles of Insulin-Resistant Patients with Polycystic Ovary Syndrome

**DOI:** 10.3390/nu9121280

**Published:** 2017-11-24

**Authors:** Mehri Jamilian, Fatemeh Foroozanfard, Elham Rahmani, Maesoomeh Talebi, Fereshteh Bahmani, Zatollah Asemi

**Affiliations:** 1 Endocrinology and Metabolism Research Center, Arak University of Medical Sciences, Arak 6618634683, Iran; Jamilian.mehri@gmail.com; 2 Department of Gynecology and Obstetrics, School of Medicine, Kashan University of Medical Sciences, Kashan 8715988141, Iran; Foroozanfard.fatemeh@gmail.com (F.F.); md_tlb2020@yahoo.com (M.T.); 3 Department of Gynecology and Obstetrics, School of Medicine, Bushehr University of Medical Sciences, Bushehr 7514633341, Iran; rahmani@bpums.ac.ir; 4 Research Center for Biochemistry and Nutrition in Metabolic Diseases, Kashan University of Medical Sciences, Kashan 8715988141, Iran; bahmani.fereshteh2@gmail.com

**Keywords:** vitamin D supplementation, hormonal profiles, insulin-resistant, polycystic ovary syndrome

## Abstract

This study was carried out to evaluate the effects of vitamin D supplementation on the metabolic profiles of insulin-resistant subjects with polycystic ovary syndrome (PCOS). This randomized double-blind, placebo-controlled trial was conducted on 90 insulin-resistant women with PCOS. Participants were randomly assigned to three groups to intake either 4000 IU of vitamin D or 1000 IU of vitamin D or placebo (*n* = 30 each group) daily for 12 weeks. Vitamin D supplementation (4000 IU), compared with vitamin D (1000 IU) and placebo, led to significant reductions in total testosterone (−0.2 ± 0.2 vs. −0.1 ± 0.6 and +0.1 ± 0.2 ng/mL, respectively, *p* = 0.02), free androgen index (FAI) (−0.06 ± 0.12 vs. −0.02 ± 0.12 and +0.004 ± 0.04, respectively, *p* = 0.04), hirsutism (−1.1 ± 1.1 vs. −0.8 ± 1.2 and −0.1 ± 0.4, respectively, *p* = 0.001) and high-sensitivity C-reactive protein (hs-CRP) (−0.7 ± 1.4 vs. −0.5 ± 0.9 and +0.5 ± 2.4 mg/L, respectively, *p* = 0.01). In addition, we found significant elevations in mean change of sex hormone-binding globulin (SHBG) (+19.1 ± 23.0 vs. +4.5 ± 11.0 and +0.7 ± 10.4 nmol/L, respectively, *p* < 0.001) and total antioxidant capacity (TAC) (+130 ± 144 vs. +33 ± 126 and −36 ± 104 mmol/L, respectively, *p* < 0.001) in the high-dose vitamin D group compared with low-dose vitamin D and placebo groups. Overall, high-dose vitamin D administration for 12 weeks to insulin-resistant women with PCOS had beneficial effects on total testosterone, SHBG, FAI, serum hs-CRP and plasma TAC levels compared with low-dose vitamin D and placebo groups.

## 1. Introduction

Polycystic ovary syndrome (PCOS) is the most common ovarian disorder associated with disturbances of reproductive, hyperinsulinemia and androgen excess in women [1]. PCOS and insulin resistance affect 5–20% [2] and 44.8–51.4% [3] of women in reproductive age worldwide. Recent evidence suggests a chronic inflammation condition in PCOS could be considered one of the potential links between PCOS, type 2 diabetes mellitus (T2DM) and cardiovascular disease [4]. In addition, the increased production of reactive oxygen species (ROS) in PCOS is related to vascular complications [5].

Hypovitaminosis D is common in women with PCOS [6]. It has been reported that vitamin D status may contribute to the progress of the metabolic disorders associated with PCOS, chiefly hyperinsulinemia and impaired glucose tolerance states [6]. Results of a meta-analysis demonstrated that vitamin D supplementation was beneficial in reducing total testosterone levels, but did not affect other androgenic profiles [7]. However, a meta-analysis study conducted by He et al. [8] indicated that vitamin D intake did not affect metabolic and hormonal profiles in PCOS women. In another meta-analysis study, vitamin D administration decreased parathyroid hormone and triglycerides levels in PCOS women, but could not influence insulin metabolism and androgens [9].

As there is evidence that vitamin D intake may improve hormonal profiles, and has anti-inflammatory and antioxidant effects, we hypothesized that vitamin D supplementation might help subjects with PCOS to control hormonal profiles, biomarkers of inflammation and oxidative stress. To our knowledge, there is scarce data on different doses of vitamin D supplementation on hormonal profiles, biomarkers of inflammation and oxidative stress in insulin-resistant patients with PCOS. The Food and Nutrition Board of the National Academy of Sciences states that 4000 IU vitamin D/day is the highest dosage with no adverse effect [10]. Substantial concern has been described about the safety of taking vitamin D at dosages higher than 1000 IU/day [10,11]. Gloth et al. [12] showed that in older populations with 25-hydroxyvitamin D concentrations < 25 nmol/L, vitamin D intakes ranged as high as 1100 IU/day. The purpose of the present study was to evaluate the effects of two different doses of vitamin D supplementation on metabolic profiles among PCOS patients.

## 2. Materials and Methods

### 2.1. Trial Design, Participants and Ethics Statements

This randomized, double-blind, placebo-controlled trial, registered in the Iranian website for registration of clinical trials as http://www.irct.ir: IRCT201604145623N75, was conducted among 90 insulin-resistant (homeostasis model of assessment-insulin resistance (HOMA-IR) > 2.5) women, aged 18–40 years old with PCOS who were referred to the Kosar Clinic, Arak, Iran, from March 2016 to December 2016. We recruited patients from 7 March 2016 till 15 March 2016 from our Referral center for PCOS in Arak. However, due to administrative problems, the current study registered as retrospective registration (20 April 2016), the intervention was started at 16 March 2016 till 15 June 2016. Therefore, we had 12 weeks of intervention for this study. Drafting the manuscript as well as data analyses was done in 3 months. All study participants were rather young, but were already obese. This investigation was carried out in accordance with the Declaration of Helsinki, and informed consent was taken from all subjects. Diagnosis of PCOS was performed according to the Rotterdam criteria [13]. Women who were pregnant during the intervention, had androgen-secreting tumors, hyperprolactinemia, thyroid dysfunction, diabetes or impaired glucose tolerance at enrollment time were excluded from the study. 

### 2.2. Study Design

Subjects were randomly assigned into three groups to take either high-dose vitamin D + metformin (4000 IU/day) or low-dose vitamin D + metformin (1000 IU/day) or vitamin D placebo + metformin (*n* = 30 each group) for 12 weeks. We used dosage of 4000 IU vitamin D based on a previous study in PCOS women [14]. Vitamin D and its placebo were manufactured by Zahravi Pharmaceutical Company (Tabriz, Iran) and Barij Essence Pharmaceutical Company (Kashan, Iran), respectively. The appearance of the placebo capsules, such as color, shape, size, and packaging, were identical to vitamin D3 capsules. To evaluate the compliance, 25(OH) vitamin D levels of participants was quantified by the enzyme-linked immunosorbent assay (ELISA) method. All subjects completed 3-day food records at weeks 0, 3, 6, 9 and 12 of the intervention. Daily macro- and micro-nutrient intakes were analyzed by nutritionist IV software (First Databank, San Bruno, CA, USA). 

### 2.3. Assessment of Anthropometric Measures

A trained staff member at the clinic did the anthropometric measurements at baseline and after the 12-week intervention (Seca, Hamburg, Germany) body mass index (BMI) was calculated as weight in kg divided by height in meters squared. 

### 2.4. Clinical Assessment

Clinical assessment included determinations of hirsutism using a modified Ferriman-Gallwey score (mFG) scoring system [15]. 

### 2.5. Biochemical Assessment

We considered hormonal profiles as primary variables, and biomarkers of inflammation and biomarkers of oxidative stress as secondary variables. Ten milliliter fasting blood samples were taken before and after the 12-week treatment at Arak reference laboratory, Arak, Iran. Serum 25-hydroxyvitamin D levels were determined by an ELISA kit (IDS, Boldon, UK) with inter- and intra-assay coefficient variances (CVs) of 4.6 to 6.7%, respectively. Serum insulin levels were measured using an ELISA kit (DiaMetra, Milano, Italy) with intra- and inter-assay CVs of 2.7 and 4.5%, respectively. Enzymatic kit (Pars Azmun, Tehran, Iran) was used to determine FPG. Insulin-resistant was considered as HOMA-IR > 2.5. Serum total testosterone with inter- and intra-assay CVs of 3.8 to 5.6% and sex hormone-binding globulin (SHBG) with inter- and intra-assay CVs of 3.3 to 9% were determined by using commercial kits (DiaMetra, Milano, Italy). Free androgen index (FAI) was calculated as the ratio of total testosterone to SHBG. Dehydroepiandrosterone sulfate (DHEAS) values were measured using an ELISA kit (Monobind, Lake Forest, CA, USA) with inter- and intra-assay CVs of 3.9 to 0.5%, respectively. Serum hs-CRP values were quantified using an ELISA kit (LDN, Nordhorn, Germany) with inter- and intra-assay CVs of 4.3 to 6.4%, respectively. The plasma nitric oxide (NO) by the Griess method, TAC by the use of the ferric reducing antioxidant power method developed by Benzie and Strain, total glutathione (GSH) using the method of Beutler et al. and malondialdehyde (MDA) levels by the thiobarbituric acid reactive substance spectrophotometric test with inter- and intra-assay CVs lower than 5% were determined. 

### 2.6. Sample Size

To determine the sample size, we considered type one error (α) of 0.05 and type two error (β) of 0.20 (power = 80%). Based on a previous study [16], we used 0.11 ng/mL as SD and 0.09 ng/mL as the difference in mean of total testosterone levels as primary variable. Based on this, we needed 25 subjects in each group. Considering a dropout rate of 5 subjects per group, we calculated we needed to have 30 subjects per group.

### 2.7. Randomization

Randomization assignment was conducted by computer-generated random numbers. Randomization and concealment of the allocations from the investigators and populations were carried out by trained staff at the clinic.

### 2.8. Statistical Analysis

We used the Kolmogrov-Smirnov test to examine the normal distribution of variables. One-way analysis of variance (ANOVA) was used to detect differences in anthropometric characteristics and dietary intakes between the three groups. To determine the effects of vitamin D supplementation on metabolic profiles, we used ANOVA test. The changes across three groups were compared using Bonferoni post hoc pair-wise comparisons. To control the effect of confounders, we adjusted all analyses for baseline values, age and baseline BMI to avoid potential bias. These analyses were conducted by analysis of covariance (ANCOVA) using general linear models. We used Pearson correlations analysis to assess between changes of metabolic profiles and changes in the 25(OH)D3 concentrations. *p* < 0.05 was considered as statistically significant. All statistical analyses were done using the Statistical Package for Social Science version 18 (SPSS Inc., Chicago, IL, USA).

## 3. Results

**Figure 1:**
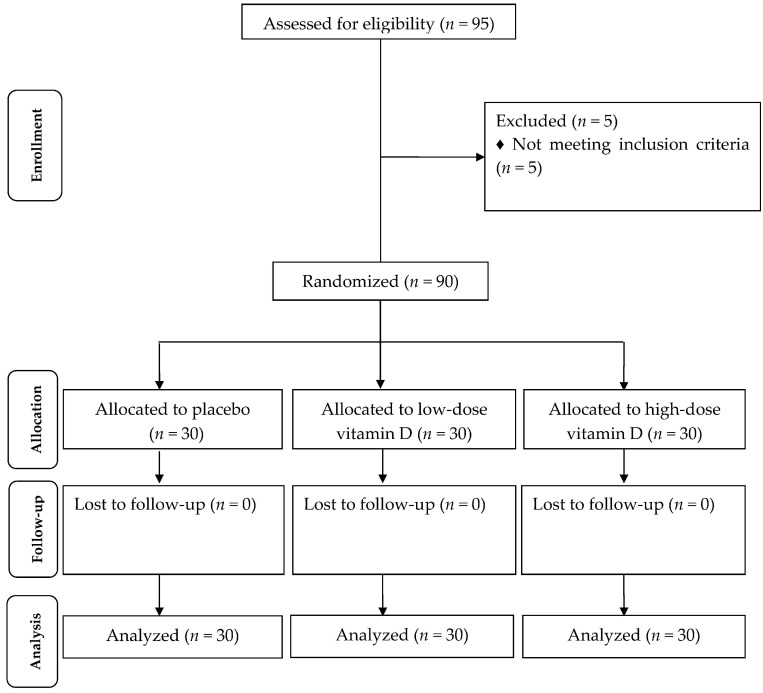
Summary of patient flow diagram.

Firstly, we invited 248 participants with PCOS (44 participants from usual attendance and 204 participants from pool); however, 112 subjects were excluded in the eligibility phase because of not meeting inclusion criteria (HOMA-IR < 2.5) and 41 others did not agree to participate. Out of 95 participants who were selected for the intervention, 5 participants were excluded from the study because of diagnosis of diabetes (Figure 1). All patients were taking the metformin tablet at the initial dose of 500 mg, which was enhanced in a stepwise manner during the first 3 weeks to incorporate the side effects until the patients were taking a total of 1500 mg/day [17]. No side effects were reported following the intake of high-dose vitamin D and metformin in patients with PCOS throughout the study.

Mean age, height, and weight and BMI at baseline and end-of-trial were not statistically different between the three groups (Table 1). Pearson correlations analysis revealed that there was no association between the changes in metabolic equivalents (MET)-h and the changes in BMI (*R*^2^ = 0.01, *p* = 0.23).

We found no significant difference in mean dietary macro- and micro-nutrient intakes between the three groups at the throughout the trial (Table 2).

Vitamin D supplementation (4000 IU), compared with vitamin D (1000 IU) and placebo, led to a significant increase in 25-hydroxyvitamin D values (+12.0 ± 2.5 vs. +5.9 ± 4.4 and +0.2 ± 0.9 ng/mL, respectively, *p* < 0.001) (Table 3). Vitamin D supplementation (4000 IU), compared with vitamin D (1000 IU) and placebo, led to significant reductions in FPG (−3.8 ± 6.9 vs. −3.2 ± 3.2 and −0.1 ± 3.6 mg/dL, respectively, *p* = 0.009), serum insulin levels (−2.3 ± 2.1 vs. −1.3 ± 3.1 and +0.1 ± 2.9 µIU/mL, respectively, *p* = 0.003), HOMA-IR (−0.5 ± 0.4 vs. −0.3 ± 0.7 and −0.1 ± 0.6, respectively, *p* = 0.004), total testosterone (−0.2 ± 0.2 vs. −0.1 ± 0.6 and +0.1 ± 0.2 ng/mL, respectively, *p* = 0.02), FAI (−0.06 ± 0.12 vs. −0.02 ± 0.12 and +0.004 ± 0.04, respectively, *p* = 0.04), hirsutism (−1.1 ± 1.1 vs. −0.8 ± 1.2 and −0.1 ± 0.4, respectively, *p* = 0.001) and hs-CRP (−0.7 ± 1.4 vs. −0.5 ± 0.9 and +0.5 ± 2.4 mg/L, respectively, *p* = 0.01). In addition, we found significant elevations in mean change of SHBG (+19.1 ± 23.0 vs. +4.5 ± 11.0 and +0.7 ± 10.4 nmol/L, respectively, *p* < 0.001) and TAC (+130 ± 144 vs. +33 ± 126 and −36 ± 104 mmol/L, respectively, *p* < 0.001) in the high-dose vitamin D group compared with low-dose vitamin D and placebo groups. We did not observe any significant effect on DHEAS, NO, GSH and MDA values following the supplementation of high-dose vitamin D compared with low-dose vitamin D and placebo groups.

When we controlled the analysis for baseline values of biochemical parameters, age and baseline BMI, hs-CRP (*p* = 0.10) became non-significant, and other findings did not alter (Table 4).

**Figure 2:**
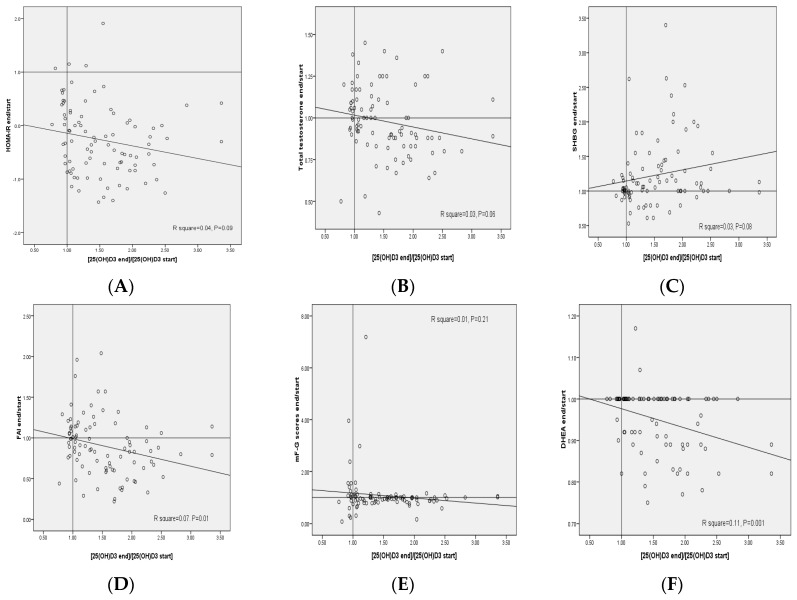

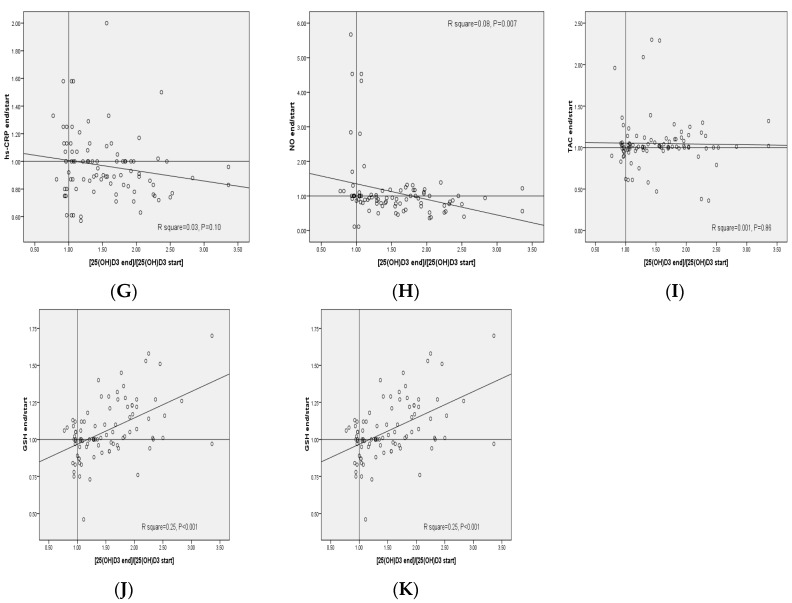
(**A**–**K**) Pearson correlation coefficients between changes of metabolic profiles and changes in the 25(OH)D3 concentrations in patients with polycystic ovary syndrome. DHEAS, dehydroepiandrosterone sulfate; FAI, free androgen index; GSH, total glutathione; HOMA-IR, homeostasis model of assessment-estimated insulin resistance; high-sensitivity C-reactive protein; mF-G, modified Ferriman Gallwey; MDA, malondialdehyde; NO, nitric oxide; SHBG, sex hormone-binding globulin; TAC, total antioxidant capacity.

The plot of the changes in FAI, serum DHEAS and plasma NO levels against the respective changes in 25(OH)D3 concentrations provided statistically significant negative correlations (*R*^2^ = 0.07, *p* = 0.01, *R*^2^ = 0.11, *p* = 0.001 and *R*^2^ = 0.08, *p* = 0.007, respectively) (Figure 2). In addition, the plot of the changes in plasma GSH levels against the respective changes in 25(OH)D3 concentrations provided statistically significant positive correlations (*R*^2^ = 0.25, *p* < 0.001).

## 4. Discussion

We found that vitamin D supplementation at a dosage of 4000 IU/day for 12 weeks to insulin-resistant women with PCOS had beneficial effects on total testosterone, SHBG, FAI, hs-CRP and TAC levels compared with 1000 IU/day of vitamin D and placebo groups, but did not affect DHEAS, NO, GSH and MDA levels. To our knowledge, data on vitamin D supplementation on hormonal profiles, biomarkers of inflammation and oxidative stress in insulin-resistant women with PCOS are scarce. It must be considered that in the current study, baseline levels of vitamin D among insulin-resistant patients with PCOS were low (~12 ng/mL). Vitamin D deficiency is highly prevalent in Iranian women due to their particular form of dressing and coverage. Earlier studies in the country have shown that PCOS is prevalent among 20% of adult women [18] and vitamin D deficiency affect 75% of women [19].

Subjects with PCOS are susceptible to hyperandrogenism, increased biomarkers of inflammation and oxidative [14,20]. The current study demonstrated that compared with 1000 IU/day of vitamin D and placebo, supplementation with 4000 IU/day of vitamin D for 12 weeks to PCOS women resulted in significant decreases in serum insulin, HOMA-IR, serum total testosterone levels, FAI and hirsutism, and a significant increase in SHBG, but did not influence DHEAS. It must be kept in mind that in the current study, no signifiant effect in HOMA-IR (or other parameters of PCOS) was observed after 12 weeks of metformin treatment in the placebo and the low-dose vitamin D groups. This may be due to lack of efficacy of metformin in our cohort. We have previously shown that vitamin D-K-calcium co-administration for 8 weeks to women with PCOS was effective in reducing free testosterone and DHEAS, but did not affect other hormonal profiles [21]. Vitamin D and calcium co-supplementation to PCOS women for 12 weeks also resulted in a significant reduction in total testosterone and androstenedione levels [22]. In addition, vitamin D and calcium co-supplementation in PCOS women could result in a better outcome in a variety of PCOS symptoms, such as menstrual regularity, and ovulation [23]. However, vitamin D supplementation for 6 months among obese vitamin D-deficient PCOS women had no significant effect on androgen levels and clinical features of hyperandrogenism [24]. In addition, two meta-analysis studies conducted by He et al. [8] and Xue et al. [9] demonstrated no evidence that vitamin D intake reduced or mitigated metabolic and hormonal profiles in PCOS women. It must be kept in mind that in our study, total testosterone levels, FAI and hirsutism significantly decreased and significantly SHBG levels that was against two previous meta-analysis studies [8,9]. This may have few reasons. The study duration may be one possible explanation for such discrepancy. Five of sixteen studies in meta-analysis by Xue et al. [9] and three of six studies in meta-analysis by He et al. [8] had a study duration lower than 3 months. The short duration of randomized controlled trials may cause non-significant effect on androgens. In addition, the subjects recruited in interventional studies had different baseline levels of vitamin D, insulin levels and HOMA-IR score. For instance, in a meta-analysis study conducted by He et al. [8], two of six studies had evaluated the effects of vitamin D supplementation on metabolic profiles in PCOS women with vitamin D deficiency. Thus, we assumed that intervention with vitamin D in the PCOS population may be important; while the beneficiary effect of vitamin D on androgens may be increased when individuals have longer duration of supplementation and baseline levels of vitamin D deficiency. In the current study, all patients had insulin resistance according to HOMA-IR criteria, whereas this was not reported in the abovementioned meta-analysis studies. On the other hand, some researchers could not observe any beneficial effect of vitamin D supplementation on glycemic control and other metabolic profiles. For example, vitamin D supplementation at a dosage of 30,000 IU once weekly for 8 weeks to populations with abnormal glucose tolerance [25], 1200 IU/day for 16 weeks to non-Western vitamin D-deficient immigrants with prediabetes [26] and 28,000 IU once weekly for 24 weeks to patients at risk for T2DM [27] did not affect glucose homeostasis parameters. Similar findings were observed following the supplementation of vitamin D at a dosage of 20,000 IU/week for 1 year in populations with prediabetes [28], a bolus oral dose of 100,000 IU cholecalciferol followed by 4000 IU cholecalciferol/day for 16 weeks in overweight or obese subjects [29] and 50,000 IU ergocalciferol/week for 12 weeks in healthy adults [30]. The different findings might be explained by different study designs, different dosages of vitamin D used as well as different populations of the study. Hyperinsulinemia may directly be associated with the development of hyperandrogenism [31], or activation of cytochrome P450c17 enzyme and the reduction of SHBG synthesis [32]. It was reported that that decreasing insulin resistance due to weight loss or metformin therapy [33] may result in a significant decrease in androgen levels. In addition, estrogen biosynthesis is regulated by vitamin D through the decrease of aromatase gene expression as well as maintaining homeostasis of extracellular calcium [33]. 

We have previously documented that the taking of calcium plus vitamin D for 8 weeks by overweight and vitamin D-deficient subjects with PCOS had beneficial effects on hs-CRP, TAC and GSH, but did not affect NO levels [20]. Vitamin D3 supplementation (200,000 IU) in elderly women with vitamin D insufficiency also decreased inflammatory factors and increased TAC levels, but did not influence MDA levels [34]. Two meta-analysis studies have reported the effects of vitamin D supplementation on CRP levels in subjects without PCOS [35,36]. In meta-analysis study conducted by Chen et al. [35], it was documented that vitamin D intake was useful in reducing hs-CRP. However, vitamin D supplementation did not affect selected inflammatory biomarkers, including CRP, tumor necrosis factor alpha and interleukin 6 in the obese and overweight subjects. In contrast with our findings, supplementation with 50,000 IU/2 weeks vitamin D3 for 4 months among adult populations with non-alcoholic fatty liver disease led to amelioration in MDA levels, but unchanged TAC values [37]. Increased biomarkers of oxidative stress may play an important role in the pathophysiology of PCOS women [38]. Less production of parathyroid hormone following supplementation of vitamin D may result in decreased production of inflammatory factors [39]. Vitamin-D may also decrease inflammatory cytokines through inhibiting nuclear factor-κB [40]. In addition, decreasing production of ROS and pro-inflammatory factors by vitamin D supplements may explain its beneficial effects on oxidative stress [41]. 

Our study had a few limitations. Firstly, in the current study, the evaluation of insulin resistance was only based on HOMA-IR. We evaluated no direct dynamic test, such as glucose tolerance test or hyperinsulinemic euglycemic clamp. Therefore, this should be taken into account in the interpretation of our findings. Another limitation of the current study was its single-center, randomized double-blind, placebo-controlled trial and ethnicity group. In addition, the use of concomitant metformin with vitamin D or placebo may affect results. However, individuals in both intervention and non-intervention groups were taking metformin; this should be considered in the interpretation of our findings. In addition, we did not evaluate the effects of vitamin D supplementation on HbA1c, follicle-stimulating hormone and luteinizing hormone. To obtain nutrient intakes of the participants, we used 3-day food diaries. This questionnaire cannot accurately reflect habitual dietary intake, as the day-to-day variation in individuals is high in most populations. Despite this, our findings about 3-day food diaries should be interpreted with caution.

## 5. Conclusions

Overall, high-dose vitamin D supplementation (4000 IU/day) for 12 weeks to insulin-resistant women with PCOS had beneficial effects in total testosterone, SHBG, FAI, hs-CRP and TAC values compared with low-dose vitamin D (1000 IU/day) and placebo groups, but unchanged DHEAS, NO, GSH and MDA values.

## Figures and Tables

**Table 1:** General characteristics of study participants ^1^.

General Measurements	Placebo (*n* = 30)	Low-Dose Vitamin D (*n* = 30)	High-Dose Vitamin D (*n* = 30)	*p* ^2^
Age (year)	25 ± 5	26 ± 5	28 ± 5	0.65
Height (cm)	161 ± 6	158 ± 6	161 ± 7	0.32
Weight at study baseline (kg)	78 ± 15	83 ± 13	81 ± 18	0.34
Weight at end-of-trial (kg)	78± 14	83 ± 13	80 ± 18	0.32
Weight change (kg)	−0.3 ± 1	−0.6 ± 2	−0.4 ± 1	0.32
BMI at study baseline (kg/m^2^)	30 ± 6	33 ± 5	31 ± 6	0.79
BMI at end-of-trial (kg/m^2^)	30 ± 6	33 ± 5	31 ± 6	0.78
BMI change (kg/m^2^)	−0.1 ± 0.5	−0.2 ± 0.6	−0.1 ± 0.5	0.78
MET-h/day at study baseline	28 ± 1	28 ± 1	27 ± 2	0.15
MET-h/day at end-of-trial	28 ± 1	28 ± 1	27 ± 2	0.24
MET-h/day change	−0.1 ± 0.3	0.009 ± 0.4	0.1 ± 0.3	0.17

^1^ Data are means ± standard deviations (SDs); ^2^ Obtained from analysis of variance (ANOVA) test; METs, metabolic equivalents.

**Table 2:** Dietary intakes of study participants throughout the study ^1^.

Nutrients	Placebo (*n* = 30)	Low-Dose Vitamin D (*n* = 30)	High-Dose Vitamin D (*n* = 30)	*p* ^2^
Energy (kcal/day)	2368 ± 233	2329 ± 272	2256 ± 268	0.24
Carbohydrates (g/day)	321.0 ± 41.0	320.8 ± 47.2	302.0 ± 46.3	0.17
Protein (g/day)	86.3 ± 13.4	85.6 ± 18.5	84.2 ± 19.0	0.89
Fat (g/day)	85.6 ± 13.5	78.8 ± 14.9	82.8 ± 14.7	0.19
SFA (g/day)	26.0 ± 5.0	25.1 ± 5.3	25.8 ± 5.5	0.78
PUFA (g/day)	26.6 ± 7.1	24.3 ± 7.1	26.0 ± 7.1	0.43
MUFA (g/day)	24.0 ± 6.3	21.4 ± 5.6	23.3 ± 6.2	0.25
Cholesterol (mg/day)	194.3 ± 73.7	213.5 ± 125.8	206.0 ± 114.9	0.78
TDF (g/day)	18.9 ± 4.2	19.6 ± 5.0	18.5 ± 4.3	0.61
Vitamin D (µg/day)	2.9 ± 1.0	2.8 ± 0.8	3.0 ± 1.0	0.76

^1^ Data are means ± SDs; ^2^ Obtained from ANOVA test; MUFAs, monounsaturated fatty acids; PUFAs, polyunsaturated fatty acids; SFAs, saturated fatty acids; TDF, total dietary fiber.

**Table 3:** Hormonal profiles, biomarker of inflammation and oxidative stress at baseline and after the 12-week intervention in insulin-resistant patients with polycystic ovary syndrome ^1^.

Variable	Placebo (*n* = 30)			Low-Dose Vitamin D (*n* = 30)			High-Dose Vitamin D (*n* = 30)			*p* ^2^
	Wk0	Wk12	Change	Wk0	Wk12	Change	Wk0	Wk12	Change	
25-OH-vitamin D (ng/mL)	12.9 ± 2.4	13.1 ± 2.5	0.2 ± 0.9	12.6 ± 3.4	18.5 ± 4.9	5.9 ± 4.4 ^a^	12.6 ± 2.7	24.6 ± 3.3	12.0 ± 2.5 ^a,b^	<0.001
HOMA-IR	3.0 ± 0.3	3.1 ± 0.7	0.1 ± 0.6	3.2 ± 0.4	2.9 ± 0.6	−0.3 ± 0.7	3.2 ± 0.4	2.7 ± 0.4	−0.5 ± 0.4 ^a^	0.004
Total testosterone (ng/mL)	1.8 ± 0.6	1.9 ± 0.6	0.1 ± 0.2	1.9 ± 0.9	1.8 ± 0.9	−0.1 ± 0.6	1.6 ± 0.7	1.4 ± 0.6	−0.2 ± 0.2 ^a^	0.02
SHBG (nmol/L)	42.9 ± 18.0	43.6 ± 16.5	0.7 ± 10.4	49.0 ± 19.1	53.4 ± 24.2	4.5 ± 11.0 ^a^	40.2 ± 10.8	59.3 ± 25.3	19.1 ± 23.0 ^a,b^	<0.001
FAI	0.17 ± 0.12	0.17 ± 0.12	0.004 ± 0.04	0.18 ± 0.18	0.16 ± 0.11	−0.02 ± 0.12	0.16 ± 0.17	0.10 ± 0.07	−0.06 ± 0.12 ^a^	0.04
mF-G scores	12.3 ± 5.2	12.2 ± 5.1	−0.1 ± 0.4	14.0 ± 3.9	13.1 ± 3.7	−0.8 ± 1.2 ^a^	13.2 ± 5.7	12.1 ± 5.3	−1.1 ± 1.1 ^a^	0.001
DHEAS (µg/mL)	1.0 ± 0.3	1.0 ± 0.3	−0.04 ± 0.3	1.3 ± 0.6	1.2 ± 0.5	−0.08 ± 0.3	1.0 ± 0.5	0.9 ± 0.4	−0.1 ± 0.2	0.54
hs-CRP (mg/L)	4.2 ± 2.2	4.6 ± 2.2	0.5 ± 2.4	4.4 ± 1.0	3.9 ± 1.1	−0.5 ± 0.9	4.6 ± 1.0	3.9 ± 1.6	−0.7 ± 1.4 ^a^	0.01
NO (μmol/L)	41.8 ± 7.2	42.3 ± 9.8	0.5 ± 8.9	39.5 ± 8.9	41.0 ± 13.9	1.5 ± 16.0	40.8 ± 3.6	42.0 ± 5.9	1.2 ± 6.7	0.94
TAC (mmol/L)	754 ± 160	718 ± 202	−36 ± 104	799 ± 93	832 ± 123	33 ± 126	742 ± 67	872 ± 123	130 ± 144 ^a,b^	<0.001
GSH (µmol/L)	657 ± 181	697 ± 187	40± 99	722 ± 121	755 ± 129	34 ± 98	734 ± 176	784 ± 237	50 ± 180	0.88
MDA (µmol/L)	2.3 ± 0.5	2.4 ± 1.1	0.1 ± 1.5	2.4 ± 1.0	2.3 ± 0.8	−0.1 ± 0.6	2.1 ± 0.9	1.9 ± 0.8	−0.2 ± 0.5	0.37

^1^ All values are means ± SDs; ^2^ Obtained from ANOVA test; ^a^ significant difference with the placebo group; ^b^ significant difference with the low-dose vitamin D group; DHEAS, dehydroepiandrosterone sulfate; FAI, free androgen index; GSH, total glutathione; HOMA-IR, homeostasis model of assessment-estimated insulin resistance; high-sensitivity C-reactive protein; mF-G, modified Ferriman Gallwey; MDA, malondialdehyde; NO, nitric oxide; SHBG, sex hormone-binding globulin; TAC, total antioxidant capacity.

**Table 4:** Adjusted changes in metabolic profiles of the patients with polycystic ovary syndrome ^1^.

Variable	Placebo Group (*n* = 30)	Low-Dose Vitamin D (*n* = 30)	High-Dose Vitamin D (*n* = 30)	*p* ^2^
25-OH-vitamin D (ng/mL)	−0.1 ± 0.5	6.0 ± 0.5	12.1 ± 0.5	<0.001
HOMA-IR	−0.03 ± 0.1	−0.3 ± 0.1	−0.5 ± 0.1	0.02
Total testosterone (ng/mL)	0.1 ± 0.1	−0.03 ± 0.1	−0.3 ± 0.1	0.002
SHBG (nmol/L)	1.0 ± 2.9	3.8 ± 3.0	19.5 ± 3.0	<0.001
FAI	0.002 ± 0.01	−0.02 ± 0.01	−0.06 ± 0.01	0.001
mF-G scores	−0.2 ± 0.2	−0.8 ± 0.2	−1.1 ± 0.2	0.002
DHEAS (µg/mL)	−0.1 ± 0.04	−0.03 ± 0.04	−0.1 ± 0.04	0.21
hs-CRP (mg/L)	0.2 ± 0.3	−0.5 ± 0.3	−0.6 ± 0.3	0.10
NO (μmol/L)	1.0 ± 1.9	1.1 ± 1.1	1.1 ± 1.9	0.99
TAC (mmol/L)	−36.0 ± 23.4	39.3 ± 23.7	124.6 ± 23.5	<0.001
GSH (µmol/L)	35.2 ± 24.3	39.7 ± 24.1	48.6 ± 24.0	0.92
MDA (µmol/L)	0.1 ± 0.2	−0.1 ± 0.2	−0.3 ± 0.2	0.27

^1^ All values are means ± SEs. Values are adjusted for baseline values, age and baseline BMI; ^2^ Obtained from ANCOVA test; DHEAS, dehydroepiandrosterone sulfate; FAI, free androgen index; GSH, total glutathione; HOMA-IR, homeostasis model of assessment-estimated insulin resistance; high-sensitivity C-reactive protein; mF-G, modified Ferriman Gallwey; MDA, malondialdehyde; NO, nitric oxide; SHBG, sex hormone-binding globulin; TAC, total antioxidant capacity.

## References

[1] Conway G., Dewailly D., Diamanti-Kandarakis E., Escobar-Morreale H.F., Franks S., Gambineri A., Kelestimur F., Macut D., Micic D., Pasquali R. (2014). ESE PCOS Special Interest Group. The polycystic ovary syndrome: A position statement from the European Society of Endocrinology. Eur. J. Endocrinol..

[2] Azziz R., Carmina E., Chen Z., Dunaif A., Laven J.S., Legro R.S., Lizneva D., Natterson-Horowtiz B., Teede H.J., Yildiz B.O. (2016). Polycystic ovary syndrome. Nat. Rev. Dis. Prims.

[3] De Paula Martins W., Santana L.F., Nastri C.O., Ferriani F.A., de Sa M.F., Dos Reis R.M. (2007). Agreement among insulin sensitivity indexes on the diagnosis of insulin resistance in polycystic ovary syndrome and ovulatory women. Eur. J. Obstet. Gynecol. Reprod. Biol..

[4] Repaci A., Gambineri A., Pasquali R. (2011). The role of low-grade inflammation in the polycystic ovary syndrome. Mol. Cell. Endocrinol..

[5] Banuls C., Rovira-Llopis S., Martinez de Maranon A., Veses S., Jover A., Gomez M., Rocha M., Hernandez-Mijares A., Victor V.M. (2017). Metabolic syndrome enhances endoplasmic reticulum, oxidative stress and leukocyte-endothelium interactions in PCOS. Metabolism.

[6] Thomson R.L., Spedding S., Brinkworth G.D., Noakes M., Buckley J.D. (2013). Seasonal effects on vitamin D status influence outcomes of lifestyle intervention in overweight and obese women with polycystic ovary syndrome. Fertil. Steril..

[7] Azadi-Yazdi M., Nadjarzadeh A., Khosravi-Boroujeni H., Salehi-Abargouei A. (2017). The effect of vitamin D supplementation on the androgenic profile in patients with polycystic ovary syndrome: A systematic review and meta-analysis of clinical trials. Horm. Metab. Res..

[8] He C., Lin Z., Robb S.W., Ezeamama A.E. (2015). Serum Vitamin D Levels and Polycystic Ovary syndrome: A Systematic Review and Meta-Analysis. Nutrients.

[9] Xue Y., Xu P., Xue K., Duan X., Cao J., Luan T., Li Q., Gu L. (2017). Effect of vitamin D on biochemical parameters in polycystic ovary syndrome women: A meta-analysis. Arch. Gynecol. Obstet..

[10] Standing Committee on the Scientific Evaluation of Dietary Reference Intakes (1997). Dietary Reference Intakes: Calcium Phosphorus, Magnesium, Vitamin, D.; and Fluoride.

[11] Marriott B.M. (1997). Vitamin D supplementation: A word of caution. Ann. Intern. Med..

[12] Gloth F.M., Tobin J.D., Sherman S.S., Hollis B.W. (1991). Is the recommended daily allowance for vitamin D too low for the homebound elderly?. J. Am. Geriatr. Soc..

[13] Rotterdam ESHRE/ASRM-Sponsored PCOS Consensus Workshop Group (2004). Revised 2003 consensus on diagnostic criteria and long-term health risks related to polycystic ovary syndrome. Fertil. Steril..

[14] Asemi Z., Foroozanfard F., Hashemi T., Bahmani F., Jamilian M., Esmaillzadeh A. (2015). Calcium plus vitamin D supplementation affects glucose metabolism and lipid concentrations in overweight and obese vitamin D deficient women with polycystic ovary syndrome. Clin. Nutr..

[15] Yildiz B.O., Bolour S., Woods K., Moore A., Azziz R. (2010). Visually scoring hirsutism. Hum. Reprod. Update.

[16] Garg G., Kachhawa G., Ramot R., Khadgawat R., Tandon N., Sreenivas V., Kriplani A., Gupta N. (2015). Effect of vitamin D supplementation on insulin kinetics and cardiovascular risk factors in polycystic ovarian syndrome: A pilot study. Endocr. Connect..

[17] Legro R.S., Arslanian S.A., Ehrmann D.A., Hoeger K.M., Murad M.H., Pasquali R., Welt C.K. (2013). Diagnosis and treatment of polycystic ovary syndrome: An Endocrine Society clinical practice guideline. J. Clin. Endocrinol. Metab..

[18] Jalilian A., Kiani F., Sayehmiri F., Sayehmiri K., Khodaee Z., Akbari M. (2015). Prevalence of polycystic ovary syndrome and its associated complications in Iranian women: A meta-analysis. Iran. J. Reprod. Med..

[19] Hassannia T., GhaznaviRad E., Vakili R., Taheri S., Rezaee S.A. (2015). High prevalence of vitamin D deficiency and associated risk factors among employed women in a sunny industrial city. Int. J. Vitam. Nutr. Res..

[20] Foroozanfard F., Jamilian M., Bahmani F., Talaee R., Talaee N., Hashemi T., Nasri K., Asemi Z., Esmaillzadeh A. (2015). Calcium plus vitamin D supplementation influences biomarkers of inflammation and oxidative stress in overweight and vitamin d-deficient women with polycystic ovary syndrome: A randomized double-blind placebo-controlled clinical trial. Clin. Endocrinol..

[21] Razavi M., Jamilian M., Karamali M., Bahmani F., Aghadavod E., Asemi Z. (2016). The Effects of vitamin D-K-calcium co-supplementation on endocrine, inflammation, and oxidative stress biomarkers in vitamin d-deficient women with polycystic ovary syndrome: A randomized, double-blind, placebo-controlled trial. Horm. Metab. Res..

[22] Pal L., Berry A., Coraluzzi L., Kustan E., Danton C., Shaw J., Taylor H. (2012). Therapeutic implications of vitamin D and calcium in overweight women with polycystic ovary syndrome. Gynecol. Endocrinol..

[23] Tehrani H.G., Mostajeran F., Shahsavari S. (2014). The effect of calcium and vitamin D supplementation on menstrual cycle, body mass index and hyperandrogenism state of women with poly cystic ovarian syndrome. J. Res. Med. Sci..

[24] Dravecka I., Figurova J., Javorsky M., Petrikova J., Valkova M., Lazurova I. (2016). The effect of alfacalcidiol and metformin on phenotype manifestations in women with polycystic ovary syndrome—A preliminary study. Physiol. Res..

[25] Wagner H., Alvarsson M., Mannheimer B., Degerblad M., Ostenson C.G. (2016). No effect of high-dose vitamin D treatment on beta-cell function, insulin sensitivity, or glucose homeostasis in subjects with abnormal glucose tolerance: A randomized clinical trial. Diabetes Care.

[26] Oosterwerff M.M., Eekhoff E.M., Van Schoor N.M., Boeke A.J., Nanayakkara P., Meijnen R., Knol D.L., Kramer M.H., Lips P. (2014). Effect of moderate-dose vitamin D supplementation on insulin sensitivity in vitamin D-deficient non-Western immigrants in the Netherlands: A randomized placebo-controlled trial. Am. J. Clin. Nutr..

[27] Moreira-Lucas T.S., Duncan A.M., Rabasa-Lhoret R., Vieth R., Gibbs A.L., Badawi A., Wolever T.M. (2017). Effect of vitamin D supplementation on oral glucose tolerance in individuals with low vitamin D status and increased risk for developing type 2 diabetes (EVIDENCE): A double-blind, randomized, placebo-controlled clinical trial. Diabetes Obes. Metab..

[28] Sollid S.T., Hutchinson M.Y., Fuskevag O.M., Figenschau Y., Joakimsen R.M., Schirmer H., Njolstad I., Svartberg J., Kamycheva E., Jorde R. (2014). No effect of high-dose vitamin D supplementation on glycemic status or cardiovascular risk factors in subjects with prediabetes. Diabetes Care.

[29] Mousa A., Naderpoor N., de Courten M.P., Teede H., Kellow N., Walker K., Scragg R., de Courten B. (2017). Vitamin D supplementation has no effect on insulin sensitivity or secretion in vitamin D-deficient, overweight or obese adults: A randomized placebo-controlled trial. Am. J. Clin. Nutr..

[30] Mitchell D.M., Leder B.Z., Cagliero E., Mendoza N., Henao M.P., Hayden D.L., Finkelstein J.S., Burnett-Bowie S.A. (2015). Insulin secretion and sensitivity in healthy adults with low vitamin D are not affected by high-dose ergocalciferol administration: A randomized controlled trial. Am. J. Clin. Nutr..

[31] Dunaif A. (1997). Insulin resistance and the polycystic ovary syndrome: Mechanism and implications for pathogenesis. Endocr. Rev..

[32] Selimoglu H., Duran C., Kiyici S., Ersoy C., Guclu M., Ozkaya G., Tuncel E., Erturk E., Imamoglu S. (2010). The effect of vitamin D replacement therapy on insulin resistance and androgen levels in women with polycystic ovary syndrome. J. Endocrinol. Investig..

[33] Pasquali R., Gambineri A., Biscotti D., Vicennati V., Gagliardi L., Colitta D., Fiorini S., Cognigni G.E., Filicori M., Morselli-Labate A.M. (2000). Effect of long-term treatment with metformin added to hypocaloric diet on body composition, fat distribution, and androgen and insulin levels in abdominally obese women with and without the polycystic ovary syndrome. J. Clin. Endocrinol. Metab..

[34] De Medeiros Cavalcante I.G., Silva A.S., Costa M.J., Persuhn D.C., Issa C.T., de Luna Freire T.L., Gonçalves M.D. (2015). Effect of vitamin D3 supplementation and influence of BsmI polymorphism of the VDR gene of the inflammatory profile and oxidative stress in elderly women with vitamin D insufficiency: Vitamin D3 megadose reduces inflammatory markers. Exp. Gerontol..

[35] Chen N., Wan Z., Han S.F., Li B.Y., Zhang Z.L., Qin L.Q. (2014). Effect of vitamin D supplementation on the level of circulating high-sensitivity C-reactive protein: A meta-analysis of randomized controlled trials. Nutrients.

[36] Jamka M., Wozniewicz M., Walkowiak J., Bogdanski P., Jeszka J., Stelmach-Mardas M. (2016). The effect of vitamin D supplementation on selected inflammatory biomarkers in obese and overweight subjects: A systematic review with meta-analysis. Eur. J. Nutr..

[37] Sharifi N., Amani R., Hajiani E., Cheraghian B. (2014). Does vitamin D improve liver enzymes, oxidative stress, and inflammatory biomarkers in adults with non-alcoholic fatty liver disease? A randomized clinical trial. Endocrine.

[38] Hyderali B.N., Mala K. (2015). Oxidative stress and cardiovascular complications in polycystic ovarian syndrome. Eur. J. Obstet. Gynecol. Reprod. Biol..

[39] Brandi L. (2008). 1α(OH)D3 One-alpha-hydroxy-cholecalciferol—An active vitamin D analog. Clinical studies on prophylaxis and treatment of secondary hyperparathyroidism in uremic patients on chronic dialysis. Dan. Med. Bull..

[40] Al-Rasheed N.M., Bassiouni Y.A., Hasan I.H., Al-Amin M.A., Al-Ajmi H.N., Mohamad R.A. (2015). Vitamin D attenuates pro-inflammatory TNF-α cytokine expression by inhibiting NF-κB/p65 signaling in hypertrophied rat hearts. J. Physiol. Biochem..

[41] Jain S.K., Micinski D. (2013). Vitamin D upregulates glutamate cysteine ligase and glutathione reductase, and GSH formation, and decreases ROS and MCP-1 and IL-8 secretion in high-glucose exposed U937 monocytes. Biochem. Biophys. Res. Commun..

